# Disinfection by Ultra-Violet Rays

**Published:** 1914-08

**Authors:** 


**Disinfection by Ultra-Violet Rays.** Friedberger and Shioji, Deutsche Med. Woch., have sterilized vaccine by this means and, by experiments on the mouths of rabbits previously infected, have shown the possibilities of this agent in the treatment of diptheria, etc., of the mouth, vagina and other cavities.

